# *RAS* screening in colorectal cancer: a comprehensive analysis of the results from the UK NEQAS colorectal cancer external quality assurance schemes (2009–2016)

**DOI:** 10.1007/s00428-017-2162-7

**Published:** 2017-06-26

**Authors:** Susan D. Richman, Jennifer Fairley, Rachel Butler, Zandra C. Deans

**Affiliations:** 1 Department of Pathology and Tumour Biology, Leeds Institute of Cancer and Pathology, St James University Hospital, Leeds, England LS9 7TF UK; 2 UK NEQAS for Molecular Genetics, Department of Laboratory Medicine, The Royal Infirmary, Edinburgh, Scotland EH16 4SA UK; 3 Cardiff and Vale UHB-Medical Genetics University Hospital of Wales, Heath Park, Cardiff, Wales UK

**Keywords:** Colorectal, Cancer, External quality assurance

## Abstract

Evidence strongly indicates that extended *RAS* testing should be undertaken in mCRC patients, prior to prescribing anti-EGFR therapies. With more laboratories implementing testing, the requirement for External Quality Assurance schemes increases, thus ensuring high standards of molecular analysis. Data was analysed from 15 United Kingdom National External Quality Assessment Service (UK NEQAS) for Molecular Genetics Colorectal cancer external quality assurance (EQA) schemes, delivered between 2009 and 2016. Laboratories were provided annually with nine colorectal tumour samples for genotyping. Information on methodology and extent of testing coverage was requested, and scores given for genotyping, interpretation and clerical accuracy. There has been a sixfold increase in laboratory participation (18 in 2009 to 108 in 2016). For *RAS* genotyping, fewer laboratories now use Roche cobas®, pyrosequencing and Sanger sequencing, with more moving to next generation sequencing (NGS). NGS is the most commonly employed technology for *BRAF* and *PIK3CA* mutation screening. *KRAS* genotyping errors were seen in ≤10% laboratories, until the 2014–2015 scheme, when there was an increase to 16.7%, corresponding to a large increase in scheme participants. *NRAS* genotyping errors peaked at 25.6% in the first 2015–2016 scheme but subsequently dropped to below 5%. Interpretation and clerical accuracy scores have been consistently good throughout. Within this EQA scheme, we have observed that the quality of molecular analysis for colorectal cancer has continued to improve, despite changes in the required targets, the volume of testing and the technologies employed. It is reassuring to know that laboratories clearly recognise the importance of participating in EQA schemes.

## Introduction

Precision medicine is now very much a key element in clinical oncology. The identification of several biomarkers along with the advent of targeted therapies has changed the landscape of cancer treatment. One example can be found in relation to colorectal cancer (CRC), where over recent years, it has become a requirement that patients with mCRC undergo *RAS* mutation screening, prior to being offered anti-EGFR therapies, as it has been shown that patients with wildtype (WT) *RAS* may gain benefit from such treatments [3, 5, 11, 14, 19], whereas those with mutated *RAS* may in fact experience detrimental effects.

Laboratories providing such molecular biomarker testing in routine practice must ensure that this work is carried out to the highest possible standards, and this should be monitored and assessed as part of a national or international external quality assurance (EQA) scheme, where possible. Indeed, this is now a requirement for laboratories in order to meet ISO15189 accreditation https://www.ukas.com/services/accreditation-services/medical-laboratory-accreditation-iso-15189/.

The results obtained in the laboratory have direct implications upon patient treatment and as such must be accurate and reproducible to provide the best patient management and avoid over-treating or denying therapy to patients.

Participation in an EQA scheme does improve the quality of molecular pathology testing, demonstrated by the decrease of the percentage of participants with genotyping errors following continual participation [7–9, 22]. Results from two CRC national EQA schemes have been recently published. The Italian *RAS* EQA scheme reported 38% of 88 participating laboratories failed the first round of their testing program, but 72% of these participants passed the second round of testing, taking the overall pass rate to almost 90% [13]. In Germany, 11/74 (14.9%) participating laboratories failed the *KRAS* quality assurance scheme set up by the German Society for Pathology in conjunction with the Federation of the German Pathologist [1].

In the Netherlands, 17 Dutch laboratories each sent 10 CRC samples to a reference laboratory for re-testing of *KRAS*, *NRAS* and *BRAF* mutation status. All mutations reported by the participating laboratories were detected by the reference laboratory, plus an additional 10 mutations. These were not originally detected due to the testing strategy employed not covering those particular mutations [6].

Furthermore, several Europe-wide schemes have been running successful EQA schemes over the past few years, including the European Society of Pathology (ESP *KRAS* EQA) [4]. In the most recently published scheme, 27% of participants incorrectly genotyped at least one of the 10 samples received as part of the scheme, and 20% of participants reported a technical error for at least one sample [17].

The United Kingdom National External Quality Assessment Service (UK NEQAS) for Molecular Genetics scheme for CRC was established in 2009. It provides assessment of the accuracy and appropriateness of laboratories’ molecular testing of colorectal cancer samples, the interpretation of the result and the clerical accuracy of the report and promotes good practice through educational comments. We report here on the data collected from all 15 of the UK NEQAS colorectal EQA schemes, from the first scheme in 2009, with 18 participants, to the most recent 2015–2016 scheme with 108 participating laboratories from both the UK and worldwide. The data analysed includes participant numbers, methodologies employed, genotyping panels and genotyping error rates.

## Materials and methods

The UK NEQAS for Molecular Genetics EQA scheme for the molecular analysis of colorectal cancer samples was first introduced in 2009. The first two assessments were provided annually and following the identification of many critical genotyping errors, subsequent schemes (2011–2012, 2012–2013 and 2013–2014) each comprised of three runs per year. As participant numbers increased and to enable the inclusion of an appeals process, this was reduced to two runs per year, from 2014 to 2015 onwards. This currently conforms to ISO/IEC 17043:2010 (http://www.iso.org).

For each EQA year, formalin-fixed, paraffin-embedded (FFPE) colorectal tumour blocks were retrieved from histopathology departments to provide nine EQA cases. The genotyping status of each block was confirmed and validated by at least two independent laboratories, using multiple testing methodologies on FFPE sections prepared throughout the blocks, prior to distribution. Multiple sections from the top, middle and lower portions of the tumour blocks were subjected to mutation status testing, to rule out tumour heterogeneity.

Participating laboratories could receive either (a) three slide-mounted 5 μm sections only, (b) two rolled 5 μm tissue sections plus a slide-mounted section or (c) three rolled 5 μm sections. Further data on this aspect from the 2014–2015 and 2015–2016 schemes is available elsewhere. (15).

From the initial EQA run until the end of the first run in 2013–2014, only *KRAS* genotyping was assessed. Run 2 of the 2013–2014 scheme introduced genotyping of *NRAS* as a pilot scheme, enabling participants to determine the standard of their *NRAS* testing, reflecting the change in clinical practice. The phasing in of a pilot gene ensured that no laboratory would be classed as a poor performer if a critical genotyping error was reported for *NRAS* during implementation of diagnostic testing. This pilot status remained in place during run 3 of 2013–2014. Laboratories are permitted to submit their genotyping results for *BRAF* and *PIK3CA* if routinely screened but were not asked to make a clinical interpretation based upon these results. Furthermore, laboratories were told not to report any additional results, outside of those requested as part of the EQA scheme.

A mock clinical case scenario was supplied with each sample, and laboratories were asked to make a clinical interpretation of the *RAS* genotyping results, in relation to the clinical scenario, and provide information as to the methodology employed for mutation detection, the sensitivity of their tests, and also on which genes were assessed.

An evaluation of each laboratory submission was made by two independent assessors against peer-ratified criteria, then each result reviewed by a panel of assessors. All submissions were anonymised so impartiality was maintained by the assessors. A maximum of two marks was awarded for genotyping accuracy, which included the correct use of HGVS nomenclature. A maximum of two marks was available for the appropriate interpretation of the result, and a maximum of two marks was also available for clerical accuracy of the report.

Any critical genotyping errors were followed up by UK NEQAS and help, support and education offered to identify and correct the cause of the erroneous result(s). Following each scheme, each laboratory was sent a tailored report, detailing how their individual laboratory had scored for each sample tested. There was also a defined period of time where any laboratories wishing to appeal their results could do so. UK NEQAS then distributed a final scheme report, detailing the correct genotyping results and stating the average scores for genotyping, interpretation and clerical accuracy across all laboratories, as well as discussing any issues arising for the EQA run. Any relevant publications were highlighted in the scheme report, to inform laboratories of current suggested testing guidelines, and implications for clinical interpretation.

## Results

### Scheme participation

**Fig. 1:**
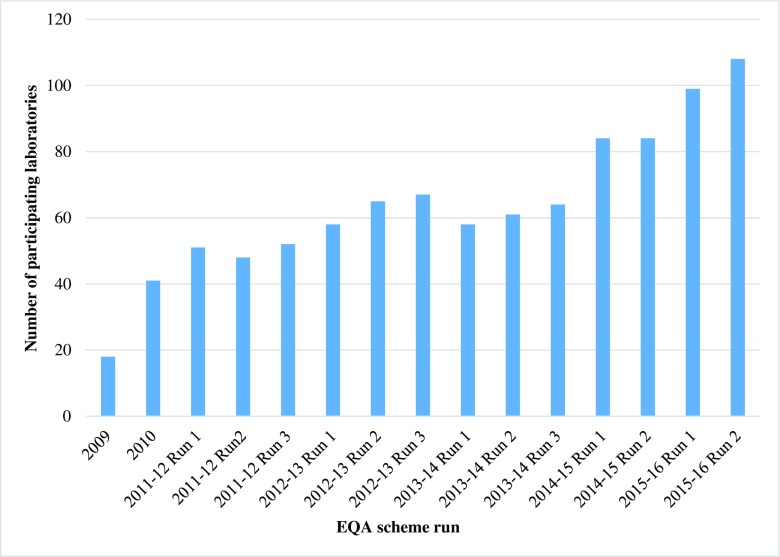
The number of participating laboratories registering for the EQA scheme each year

The number of participating laboratories rose steadily from the first EQA scheme, in 2009 (Fig. 1). The most marked increase was between the third run of 2013–2014 and the first run of 2014–2015, with an additional 20 laboratories participating. Overall, there has been a sixfold increase in the number of laboratories taking part in the scheme, since its inception. In the 2009 scheme, 47% were UK-based and 53% were international, whereas in the most recent run, as numbers increased, these figures were and 29 and 71%, respectively.

### Mutation detection methodology

The method, or in some cases, multiple methodologies used by participating laboratories was reviewed in detail for the three most recent EQA years (2013–2014, 2014–2015 and 2015–2016), thus providing data on seven consecutive runs. The total number of uses for each particular methodology was determined for each EQA year, and then this total used to determine the ranking order.

Table 1 shows the three most common methodologies employed for mutation screening of *KRAS*, *NRAS*, *BRAF* and *PIK3CA*. One of the most striking observations is the significant decrease in the usage of the Roche cobas® platform, possibly reflecting its limited coverage of mutation hotspots and the lack of ability to differentiate between mutations detected. Pyrosequencing and Sanger Sequencing still remain popular platforms, but the use of next generation sequencing (NGS) has significantly increased, becoming the most commonly used methodology for *BRAF* and *PIK3CA* screening and the 2nd and 3rd most common for *KRAS* and *NRAS*, respectively.

**Table 1:** The three most commonly used screening methodologies for mutation screening of *KRAS*, *NRAS*, *BRAF* and *PIK3CA* in the last three EQA years

Scheme year/gene	Most common methodology	Second most common methodology	Third most common methodology
2013–2014			
*KRAS*	Roche cobas®	Pyrosequencing	Sanger sequencing
*NRAS*	Pyrosequencing	Sanger sequencing	Mass spectrometry
*BRAF*	Roche cobas®	Pyrosequencing	Sanger Sequencing
*PIK3CA*	Roche cobas®	Mass spectrometry (Sequenom)	Pyrosequencing
2014–2015			
*KRAS*	Sanger sequencing	Roche cobas®	Pyrosequencing
*NRAS*	Sanger sequencing	Pyrosequencing	NGS
*BRAF*	Sanger sequencing	Pyrosequencing	NGS
*PIK3CA*	NGS	Sanger sequencing	^a^
2015–2016			
*KRAS*	Sanger sequencing	NGS	Pyrosequencing
*NRAS*	Pyrosequencing	Sanger sequencing	NGS
*BRAF*	NGS	Pyrosequencing	Sanger sequencing
*PIK3CA*	NGS	Sanger sequencing	Mass spectrometry (Sequenom)

^a^Pyrosequencing, Roche cobas® and mass spectrometry (Sequenom) all equally place third

### Mutation detection testing combinations

There has been a marked decrease in the percentage of laboratories testing only *KRAS*, and an increase in the percentage testing both *KRAS* and *NRAS*. Overall, there has been a trend towards an increase in testing the combination of *KRAS* and *NRAS* and *BRAF*, and also an increase in the testing of *KRAS*, *NRAS*, *BRAF* and *PIK3CA*. No laboratories in the 2015–2016 scheme were reporting the combination of *KRAS* plus *BRAF*, whereas in 2013–2014, this figure was 33.9 and 26.7% of laboratories respectively for runs 1 and 2 (see Table 2).

**Table 2:** Percentage of laboratories performing each of the genotyping testing combinations. As several laboratories employ more than one technology, the columns may exceed 100%

Genotyping testing combination	2013–2014	2013–2014	2013–2014	2014–2015	2014–2015	2015–2016	2015–2016
	Run 1 *n* = 58	Run 2 *n* = 61	Run 3 *n* = 64	Run 1 *n* = 84	Run 2 *n* = 84	Run 1 *n* = 99	Run 2 *n* = 108
*KRAS* only	49.2	38.3	23.8	14.3	10.7	11.2	8.2
*KRAS* + *NRAS*	0	1.7	12.7	27.4	33.3	40.8	34.7
*KRAS* + *NRAS* + *BRAF*	0	11.7	31.7	31.0	31.0	23.5	36.7
*KRAS* + *NRAS* + *BRAF* + *PIK3CA*	0	16.7	22.2	20.2	21.4	22.4	30.6
*KRAS* + *NRAS* + *PIK3CA*	0	1.7	1.6	1.2	1.2	1.0	0
*KRAS* + *BRAF* + *PIK3CA*	16.9	3.3	1.6	2.4	1.2	1.0	1.0
*KRAS* + *BRAF*	33.9	26.7	6.3	3.6	1.2	0	0

### Genotyping errors

**Fig. 2:**
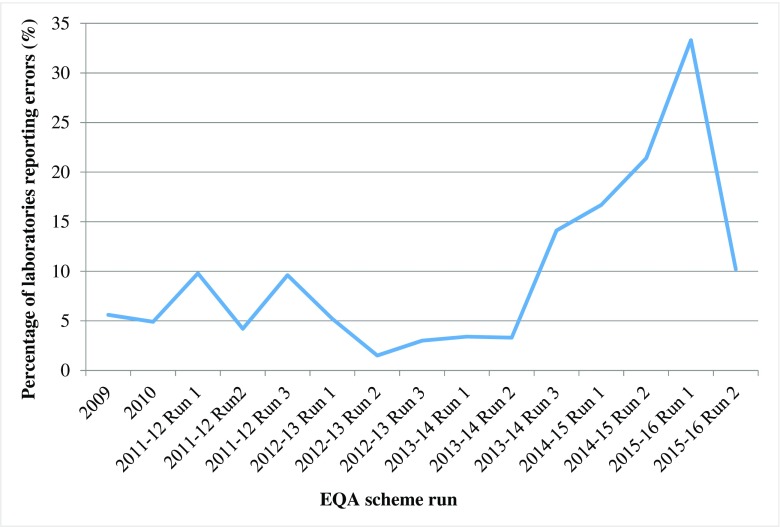
The percentage of laboratories in each scheme run, reporting a genotyping error in one of both of the *RAS* genes

For each EQA year, the total number of laboratories reporting errors in *RAS* (*KRAS* and *NRAS*) genotyping was determined (Fig. 2). This figure remained below 10% from the initial scheme in 2009, up to, and including Run 2 of the 2013–2014 scheme. The number of laboratories reporting *RAS* genotyping errors peaked at 33.3% in the initial run of the 2015–2016 scheme but dropped back down to 10.2% in the subsequent run.

**Fig. 3:**
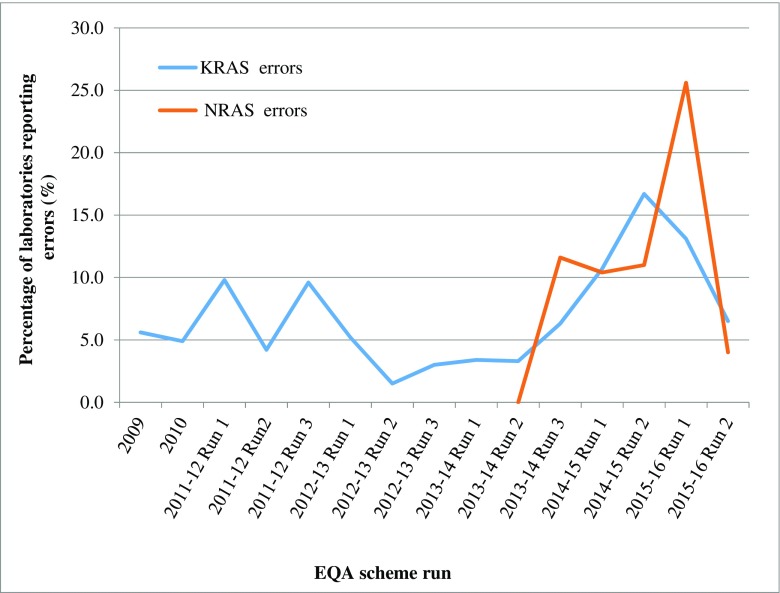
Percentage of laboratories with *KRAS* and *NRAS* genotyping errors. (*NRAS* was only introduced into the EQA scheme in the second run of 2013–2014)

Figure 3 shows the data when the *KRAS* and *NRAS* genotyping errors were analysed separately. For *KRAS*, the percentage of laboratories reporting genotyping errors remained below 10% from the first scheme in 2009 up to and including Run 3 of the 2013–2014 scheme. The rise to 16.7% in 2014–2015, corresponded with a significant increase in the number of laboratories participating in the scheme (64 in 2013–2014 Run 3, and 84 in 2014–2015 Runs 1 and 2), and furthermore, the majority of laboratories reporting incorrect *KRAS* genotypes were new participants to the scheme. The percentage reporting errors fell during the 2015–2016 schemes to 6.5% in the most recent run. When *NRAS* was introduced as a pilot scheme, in Run 2 of the 2013–2014 scheme, all of the 19 laboratories who reported a *NRAS* genotyping result reported it correctly. Over the next three runs, the percentage reporting incorrect *NRAS* genotype results increased but remained relatively stable between 10.4 and 11.6% but peaked at 25.6% in the first run of the 2015–2016 scheme, finally falling to 4% in the final run analysed. Unlike with the *KRAS* genotyping errors, the majority of *NRAS* genotyping errors in the first 2015–2016 scheme could not be attributed to new participating laboratories.

Table 3 shows the type of genotyping errors being reported by laboratories, across all EQA runs, for *KRAS*. By far, the most common error was the reporting of a false negative result, in samples where a validated mutation had been identified. The 2014–2015 scheme and Run 1 of the 2015–2016 scheme showed an increase in the number of errors being reported. In each case, there were laboratories with multiple genotyping errors.

**Table 3:** Types of genotyping errors reported in *KRAS* testing

EQA year and Run	Number of laboratories reporting genotyping errors (%)	Total number of errors reported	False positive results reported	False negative results reported	Incorrect mutation reported	^a^Extra mutation reported (not present)
2009	1 (5.5)	1	0	1	0	0
2010	2 (4.9)	5	1	2	2	0
2011–2012 Run 1	5 (9.8)	8	0	7	1	0
2011–2012 Run 2	2 (4.2)	8	1	1	6	0
2011–2012 Run 3	5 (9.6)	5	0	3	2	0
2012–2013 Run 1	3 (5.2)	4	2	2	0	0
2012–2013 Run 2	1 (1.5)	1	1	0	0	0
2012–2013 Run 3	2 (3.0)	2	0	2	0	0
2013–2014 Run 1	2 (3.4)	3	1	2	0	0
2013–2014 Run 2	2 (3.3)	2	0	2	0	0
2013–2014 Run 3	4 (6.3)	4	0	2	2	0
2014–2015 Run 1	9 (10.7)	15	8	6	1	0
2014–2015 Run 2	14 (16.7)	16	2	12	1	1
2015–2016 Run 1	13 (13.1)	25	8	11	6	0
2015–2016 Run 2	7 (6.5)	11	8	1	0	2

^a^Extra mutations differed to a false positive result, as these mutations were detected in addition to the mutation actually present in the sample, rather than being detected in a wildtype sample, which was classed as a false positive result

Table 4 shows the type of genotyping errors made by laboratories for *NRAS*. The large increase in Run 1 of 2015–2016 in the number of false negatives was partly a result of several laboratories using a TIBMOLBIOL Lightmix kit, which failed to detect the *NRAS* mutation. No other correlations were seen between the methodologies used and genotyping errors reported.

**Table 4:** Types of genotyping errors seen in *NRAS* testing

EQA year and Run	Number of laboratories reporting genotyping errors (%)	Total number of errors reported	False positive results reported	False negative results reported	Incorrect mutation reported	Extra mutation reported (not present)
2013–2014 Run 2	0	0	0	0	0	0
2013–2014 Run 3	5 (11.6)	5	1	4	0	0
2014–2015 Run 1	7 (10.4)	8	5	1	2	0
2014–2015 Run 2	8 (10.9)	8	4	3	1	0
2015–2016 Run 1	22 (25.6)	29	2	23	4	0
2015–2016 Run 2	4 (4.0)	4	1	1	0	2

### Interpretation and clerical errors

Not all laboratories submitted a specific clinical interpretation, in addition to the genotyping results. For those laboratories that did, each was expected to additionally report the reference sequence for each gene tested; the assay sensitivity (i.e., the percentage of mutant DNA that could be detected in a background of wild type DNA); a statement indicating that an assessment of the tumour had been made, and the percentage of tumour cells in the assessed area or a statement indicating that no assessment was made; the methodology employed and the scope of each assay (i.e., which mutations could be detected and in which codons or exons).

The most common errors made, which each resulted in the loss of 0.5 marks, were a lack of a gene reference sequence being stated and a statement of assay sensitivity, which was of particular importance in cases which were wild type for all assays performed. Marks were seldom deducted for clerical accuracy errors, although occasionally a typographical error in the patient name or date of birth was present, and in which case, appropriate deductions were made. A total of 2.0 marks were available for both interpretation and clerical accuracy. Table 5 shows the mean scores in both categories for each scheme year.

**Table 5:** The mean interpretation and clinical accuracy scores for participating laboratories across all scheme years. A maximum of 2.00 marks was available for each category

EQA year; run	Mean interpretation score (max 2.00)	Mean clinical accuracy score (max 2.00)
2009	Not marked	Not marked
2010	1.82	1.99
2011–2012; Run 1	1.44	1.93
2011–2012; Run 2	1.68	1.93
2011–2012; Run 3	1.84	1.98
2012–2013; Run 1	1.90	1.98
2012–2013; Run 2	1.92	1.98
2012–2013; Run 3	1.84	1.98
2013–2014; Run 1	1.93	1.98
2013–2014; Run 2	1.98	1.99
2013–2014; Run 3	1.97	2.00
2014–2015; Run 1	1.90	1.99
2014–2015; Run 2	1.93	1.98
2015–2016; Run 1	1.93	1.99
2015–2016; Run 2	1.89	1.99

## Discussion

We have now entered the era of personalised medicine with biomarker mutation status becoming an essential requirement, prior to the prescribing of particular targeted therapies. It has been long established that mutated *KRAS* is a negative predictive biomarker of response to Panitumumab [3] and Cetuximab [5, 20]. More recently, guidelines have been updated to reflect the findings of several studies, which showed that activating mutations outside of just *KRAS* exon 2 were also predictive of response to anti-EGFR therapies [10, 21].

The European Medicines Agency (EMA) now state that patients must be WT for exons 2, 3 and 4 of both *KRAS* and *NRAS*, prior to therapy. In the UK, the recommendation that *KRAS* codons 12–13, 59, 61, 117 and 146 and *NRAS* 12–13, 59 and 61 has been proposed by the Association of Clinical Pathologists Molecular Pathology and Diagnostic Group [23].

As the requirement for extended testing panels increases to influence patient management, there becomes the need to ensure that such testing is accurate across all targets tested. Since 2009, the UK NEQAS for Molecular Genetics Colorectal cancer EQA scheme has been providing assessments and has tracked the changes employed by laboratories.

The increased number of genes to be tested testing brings with it the inevitable need for larger quantities of DNA for molecular testing unless methods are changed to allow parallel testing. We have seen a shift in the most commonly used testing methodologies, reflecting this requirement, with more laboratories employing more sensitive NGS techniques which also enable multiple genes and/or gene regions to be tested at the same time. In the most recent run, 25 laboratories reported using NGS for both *KRAS* and *NRAS* testing, making it the most common method of screening. Similarly, 23 and 18 laboratories stated that NGS was used to genotype *BRAF* and *PIK3CA*, respectively. Although Sanger Sequencing and Pyrosequencing still remain the next most popular choices, as testing panels expand to incorporate additional genes, one may assume that the number of laboratories able to still employ these methods will decrease, and this is indicated in the increasing number of laboratories participating in the UK NEQAS NGS technical somatic EQA scheme.

Since the first run of the 2013–2014 scheme, there has been a significant decrease in the number of laboratories reporting only the *KRAS* mutation status, with a drop from 49.1 to just 8.2%. It is encouraging to see that laboratories are realising the need to test additional genes. In run 2 of the 2013–14 scheme, when *NRAS* genotyping was introduced as a pilot scheme, only 1.7% of laboratories reported an *NRAS* genotyping result. This increased to 40.8% in the first run of the 2015–2016 scheme but fell to 34.7% in the second run. This slight decrease was a result of the increase in the number of new participating laboratories with only *KRAS* testing employed and *NRAS* testing not yet implemented.

There has been an increase in the percentage testing *KRAS*, *NRAS* and *BRAF*, up to 36% in the most recent scheme run. Mutated *BRAF* is a poor prognostic marker [15, 16, 18]; however, the predictive significance still remains controversial, and thus *BRAF* genotyping currently is not a requirement under UK recommendations, ASCO guidelines or EMA guidelines [2, 23]. The prognostic significance and relationship with microsatellite instability (MSI) status is most likely what drives clinical laboratories to currently perform *BRAF* genotyping.

Almost one-third (30.6%) of laboratories carried out genotyping of *KRAS*, *NRAS*, *BRAF* and *PIK3CA* in the most recent scheme. This was an increase of just over 8% on the previous run and is likely the result of the increased use of NGS testing panels, which incorporate these genes in addition to *KRAS*, *NRAS* and many others. This is the reason for UK NEQAS to include multiple genes into the assessment to provide participants with a measure of the accuracy of this testing along with the assessment of the clinically actionable genes.

Genotyping errors, whether false positive, and thus potentially depriving patients of therapy, or false negative, and potentially exposing patients to treatments which will provide no benefit, and may in fact be detrimental [11, 12], were reported at varying levels across all EQA runs. Run 2 of 2013–2014 saw only 1.5% of the 65 participating laboratories reporting an incorrect genotype, but this figure peaked at 33.3% in the first run of 2015–2016. Thirteen of the 99 laboratories reported a total of 23 *KRAS* genotyping errors, and 33 of the 86 laboratories carrying out *NRAS* mutation screening, reported a total of 29 errors. Laboratories reporting errors in *NRA*S during the second and third run of 2013–2014 were not considered ‘poor performers’ as for these two runs only, the *NRAS* scheme was given pilot status. One reason for this large increase in the number incorrect NRAS genotyping errors was the use by several laboratories of a commercial kit, not designed to identify the particular *NRAS* mutation, carried by one of the tumours; c.181C > A p.(Gln61Lys). This resulted in a significant increase in the number of false negative results.

It is worth noting that each sample is assessed in the reference laboratories for tumour cell content. None of the samples used in the EQA scheme had a tumour cell content of less than 20%, thus removing this as a reason for false negative results. Several laboratories state the mutant allele frequencies within their reports, and these are above the sensitivity levels of all technologies employed by participating laboratories.

In general, those laboratories choosing to submit a clinical interpretation do so with a high degree of accuracy. There was only one scheme run where the mean score dropped below 1.50/2.00. During this particular run, only two (of the three) cases were marked, which clearly impacted upon the mean interpretation score.

When comparing the overall performance of laboratories taking part in the UK NEQAS EQA scheme, with those of other comparable schemes, it would appear that participants in this scheme do substantially better. This may be due in part, to the obvious shift we have seen, to more sensitive testing technologies, such as NGS. Participants have responded positively to feedback given by the scheme and have taken corrective action to previous errors. Furthermore, our data show that there tends to be a small number of laboratories, making multiple errors, which has a lessening impact on the overall scheme results, given the large numbers of participating laboratories in the UK NEQAS scheme.

This EQA scheme has been running for 8 years, expanding almost every year, as increasingly laboratories realise the necessity and indeed benefits of scheme participation. Continued scheme participation will ensure continued validation of methodologies which may require modification over time, to cope with the ever-increasing demands placed on laboratories for additional biomarker testing. EQA schemes such as this will remain critical to ensuring accurate genotyping and thus guiding personalised medicine.
